# Efficacy and safety of fire needle combined with cupping for acute herpes zoster and postherpetic neuralgia: A protocol for systemic review and meta-analysis

**DOI:** 10.1097/MD.0000000000032251

**Published:** 2022-12-16

**Authors:** Shijie Huang, Yipeng Xu, Zhengqi Pan, Zimeng Li, Rui Luo, Maogui Yu, Wuyu Li, Hanghang Liu, Jie Wu

**Affiliations:** a Acupuncture and Moxibustion School Chengdu University of Traditional Chinese Medicine, Chengdu, Sichuan, China; b State Key Laboratory of Oral Disease and National Clinical Research Center for Oral Diseases and Department of Oral Maxillofacial Surgery, West China Hospital of Stomatology, Sichuan University, Chengdu, Sichuan, China; c Hospital of Chengdu University of Traditional Chinese Medicine, Chengdu, Sichuan, China.

**Keywords:** cupping, fire needle, herpes zoster, post-herpetic neuralgia

## Abstract

Herpes zoster and post-herpetic neuralgia showed an increasing incidence during past two decades. Most of herpes zoster and post-herpetic neuralgia patients suffered from pain, anxiety, and depression. Fire needle combined with cupping is becoming a popular way to relieve the pain caused by herpes zoster and decrease the incidence of post-herpetic neuralgia. In this study, we aim to investigating the efficacy and safety of fire needle combined with cupping for the treatment of acute herpes zoster and postherpetic neuralgia (PHN).

**Methods:**

The literature search will be carried out in following databases: PubMed/MEDLINE, EMBASE, the Cochrane Central Register of Controlled Trials, China National Knowledge Infrastructure, Chinese Biomedical Literature Database and Wanfang Data. Published and unpublished controlled trials compared fire needle combined with cupping to other treatments for acute herpes zoster or PHN will be included. Data from eligible studies will be extracted by 2 independent reviewers. Different scales will be used to assess the risk of bias based on the study design. Pain intensity and PHN are primary outcomes. The final effect size will be reported using 95% confidence interval at 0.05 significance level.

**Discussion:**

This review will provide certain evidence to compare the efficacy and safety of combined acupuncture and cupping with guideline recommended drug or nerve block therapy for the treatment of herpes zoster and post-herpetic neuralgia. It will potentially provide more clinical suggestions and guidelines for health care professionals, policymakers, and researchers.

## 1. Introduction

Herpes zoster (HZ), also known as Shingles, is one of the most common skin infection diseases caused by the varicella-zoster virus.^[1]^ The virus usually stays inactive near spinal cord or brain in the nerve tissues after the first time infected, usually during childhood, and this virus can reactivate years later, causing HZ accompanied by severe neuralgia.^[2,3]^ Postherpetic neuralgia (PHN) usually appears after the patient’s herpes has healed near the skin lesion, it is a kind of refractory neuralgia that can last for a long time after the onset of HZ.^[4,5]^ The incidence of HZ during 1994 and 2018 increased from 2.86% to 5.79%, and the percentage of patients with HZ that developed PHN also showed an increasing trend from 1994~2006 to 2007~2018 in North America, Europe, and Asia-Pacific.^[4]^ It was reported that the pain related to HZ and PHN has significant effect in the quality of life of the affected patients, around 45% of these patients may suffer from anxiety, depression, and lack of concentration.^[6]^

HZ is usually considered to be a self-limiting disease in immunocompetent patients, antiviral therapies was recommended to hasten healing time while paracetamol/acetaminophen was used for significant pain management over 50 years.^[7]^ However, there is no evidence showed that antiviral therapies has any positive effect on reducing the incidence of PHN.^[8]^ In addition to non-steroidal anti-inflammatory drugs, glucocorticoid, nerve-blocking therapy, mental and psychological intervention were also used for HZ and PHN related pain relief.^[9–11]^ It is noteworthy that all these treatments shows unstable effect in pain management, and may increase the renal burden and impact the immune system, especially for those with renal insufficiency or immunodeficiency.

Recently, acupuncture, especially fire needle, has been reported to be an effective method for pain relief during past few decades, and it could provide notable benefit for the patients in the acute stage of HZ and PHN.^[12]^ Acupuncture could stimulate acupuncture points to relieve local pain and spasm, and then promote local blood circulation and dredge the meridians. Besides, acupuncture was also recommended as Chinese clinical practice guidelines for HZ and PHN by the Chinese Consensus of Diagnosis and Treatment of HZ and PHN. Moreover, acupuncture are frequently used combined with thermotherapy in clinical practice.^[12,13]^ Fire needle is one of the most common acupuncture that used for the treatment of acute HZ and PHN, several studies showed that fire needle could significantly reduce the incidence of PHN.^[14]^ Additionally, cupping therapy was also found to effectively relieve pain by promoting local microcirculation and decreasing the expression of pain-causing substances.^[15]^ Nowadays, it is becoming popular combining fire needle and cupping together for pain management in China. Increasing studies showed fire needle combined with cupping treatment could remarkably enhance the efficacy of fire needle and dramatically reduce HZ and PHN related pain, shorten the pain duration and decrease the incidence of PHN.^[12]^

Nevertheless, the superiority of fire needle plus cupping in the treatment of HZ and PHN is still not clear when comparing to guideline drug, block therapy or fire needle treatment only. To acquire a comprehensive understanding of the efficacy and safety of fire needle combined with cupping therapy when treating with HZ and PHN patients, we conducted this systematic review and meta-analysis, in which we aimed to providing suggestions as well as guidance for clinical practice.

## 2. Methods and analysis

This review was conducted in coincidence with previous developed protocol that registered on the National Institute of Health Research Database (CRD42022300076). This review was carried out following the Preferred Reporting Items for Systematic Reviews and Meta-Analyses Protocols guidelines.^[16]^

### 2.1. Inclusion criteria

a) Types of studies: Randomized-controlled studies (RCTs), non-randomized-controlled-studies (NRSs), cohort studies and case-control studies.
b) Patients: Adults with acute HZ (<7 days from onset), or adults that diagnosed as PHN and suffered from sustained pain for >3 months.^[17]^
c) Interventions: Fire needle combined with cupping.
d) Control: guideline recommended drug or nerve block therapy, fire needle only, other acupuncture techniques such as electroacupuncture and moxibustion.
e) Outcomes:
  1) Primary outcomes: HZ or PHN related pain intensity measured using Visual Analogue Scale, Verbal Rating Scale, McGill pain score, or other rating scales (e.g., Faces Pain Scale); duration to relief pain; PHN incidence (Pain that persists 4 weeks after HZ resolves);
  2) Secondary outcomes: quality of life measures; duration of lesion healing; therapeutic effective rate on the basis of Chinese that guideline proposed by State Administration of Traditional Chinese Medicine; incidence and severity of any other adverse effects.

### 2.2. Exclusion criteria

1. Case report, case series, and reviews.
2. Abstract or conference with incomplete limited data.
3. Patients with HZ complications including HZ meningoencephalitis, Ramsay Hunt syndrome, zoster opthalmicus (combined with conjunctivitis, keratitis or scleritis), visceral or disseminated zoster, Vernet syndrome, and concomitant bacterial infections.
4. Patients used HZ vaccine as adjunct to intervention or control treatment.

### 2.3. Search strategy

**Figure 1.**
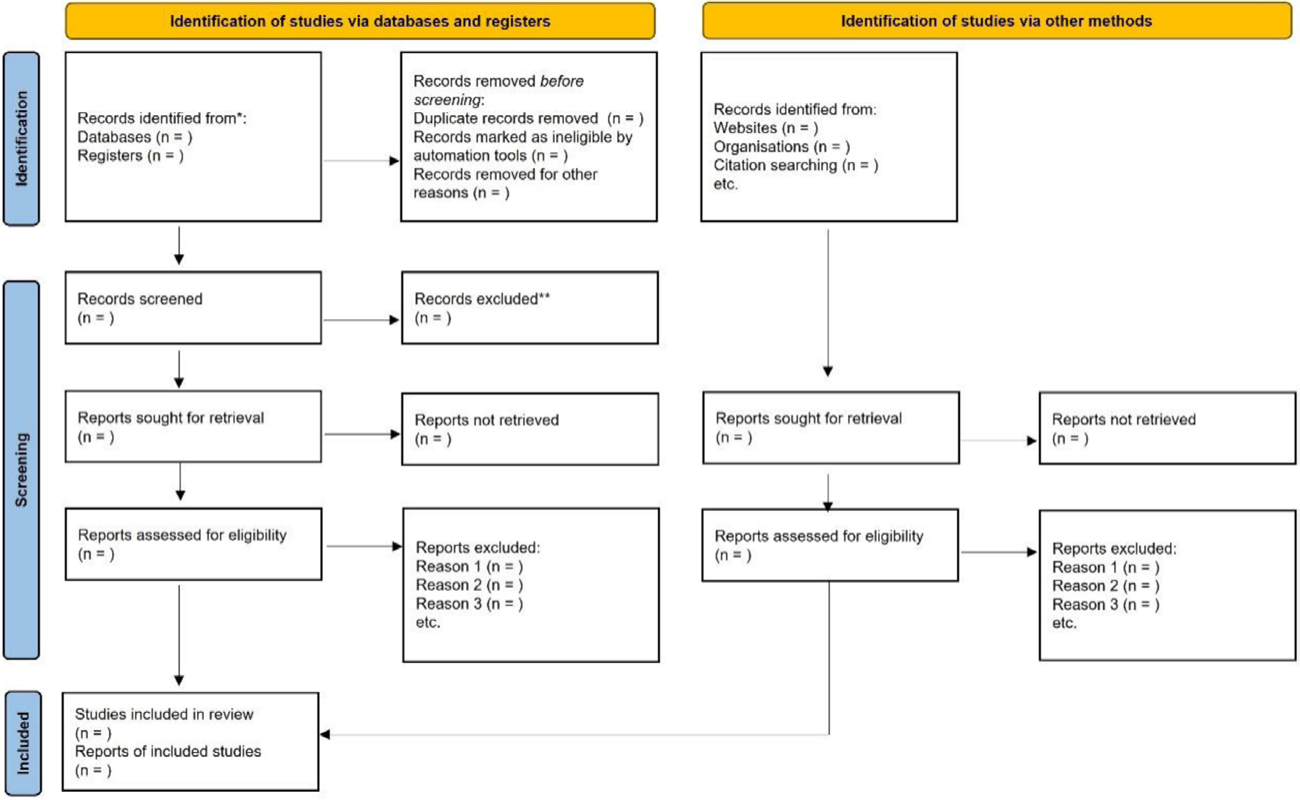
Flow diagram of study selection for this systematic review and meta-analysis.

The literature search will be carried out in following databases: PubMed or MEDLINE (via OVID), EMBASE (via OVID), the Cochrane Central Register of Controlled Trials, China National Knowledge Infrastructure, Chinese Biomedical Literature Database and Wanfang Data. Sources of unpublished documents and ongoing trails to be searched in Sciencepaper Online, Opengray, World Health Organization International Clinical Trials Registry Platform and other medical societies/associations. Additional studies will be identified through the references of included studies. The flow chart for this systematic review is described in Figure 1.

A separate searching strategy will be developed for each database with following keywords and related terms: (fire needle OR fire acupuncture OR red hot needle OR burn needle) AND (cupping OR wet cupping OR dry cupping OR pricking cupping OR moving cupping OR flash cupping) AND (HZ OR shingles OR postherpetic neuralgia OR PHN OR herpetic neuralgia).

Anticipated date: March 2023 to August 2023.

### 2.4. Data extraction

All the retrieved records will be imported to EndNote X8. After removing duplicate studies, title and abstract will be screened and checked independently at least by 2 reviewers and the full text of identified researches will be further assessed for eligibility. Following characteristics will be collected: author name, publication year, study design, gender distribution, sample size, publishing region, follow-up duration, clinical diagnosis, interventions, major outcomes and measures of interest. Any discrepancies on primary article data collection or critical appraisal will lead to consult the third reviewer.

### 2.5. Risk of bias assessment

According to different types of study design, corresponding scales will be applied to evaluate the risk of bias by 2 independent reviewers. Cochrane criteria, Methodological Index for Non-Randomized Studies and Newcastle–Ottawa scale will be used for RCTs, cohort (or case control) studies and NRSs evaluation, respectively.

### 2.6. Assessing certainty in the findings

The methodological quality of each evidence will be assessed via the 2009 Oxford Centre for Evidence-based Medicine Levels of Evidence or Grading of Recommendations, Assessment, Development, and Evaluation approach.^[18]^

### 2.7. Data analysis

All the statistical analyses will be done by RevMan software (version: 5.3) or STATA Analytical Software (version 14, StataCorp). For dichotomous variables, odds ratio will be used for data synthesis from case-control studies, while risk ratio using the Mantel–Haenszel test will be used for RCTs and NRSs. In contrast, weighted mean difference will be applied for continuous variables using inverse variance method if the combined measures were numerically similar. If not, standard mean difference will be applied instead. All the pooled effect will be displayed using 95% confidence interval with a significance level of 0.05. Missing data will be retrieved by contacting with related corresponding authors. Those studies that are not eligible for meta-analysis will be discussed using a narrative synthesis approach.

Chi-square test (*I*^2^ statistic) will be used for the evaluation of data heterogeneity, *I*^2^ < 10% will be considered with low heterogeneity. In order to detecting potential clinical heterogeneity, meta-regression or Galbraith plot will be carried out. Subgroup analysis will be applied according to study design, age of included patients, intervention or control characters to minimize related clinical heterogeneities. Random effects model will be used for all the meta-analysis that with remarkable heterogeneity (25%). Sensitivity analysis will be conducted by sequential removal of each single study from the analysis.

Publication bias will be assessed by funnel plot, Egger’s weighted liner regression test or Harbord test at a 5% significance level for the meta-synthesis containing >10 studies. Discrepancies between reviewers will be resolved by consulting the third reviewer. Trim and fill analysis will be done for the synthesis with significant publication bias, and adjusted effect sizes will be reported.

## 3. Discussion

HZ and PHN showed an increasing incidence during past two decades, both HZ and PHN will bring remarkable negative impact on the patients’ quality of life, daily activities and economic well-being of patients.^[3,19,20]^ Although drug or nerve block therapy was recommended for the pain management in HZ and PHN patients nowadays, approximately half patients may still not get sufficient and satisfactory pain relieve.^[21]^ Besides, few worldwide suggested front-line therapy for PHN including antiepileptics will still bring few side-complications.

Acupuncture and cupping have a prominent place Chinese traditional medicine and has a theatrical history of >2000 years. Recently, fire needle plus cupping was suggested for the treatment of HZ an PHN in China, but no high quality synthesis study was conducted. Therefore, we conducted this systematic review and meta-analysis, hoping to provide more clinical suggestions and guidelines for the treatment of HZ and PHN.

## 4. Strengths and limitations of this study

This is the first systematic review and meta-analysis protocol designed to investigate the efficacy and safety of fire needle combined with cupping for the treatment of both acute HZ and PHN. The protocol is reported according to Preferred Reporting Items for Systematic Reviews and Meta-Analyses Protocols checklist, and we provided detailed heterogeneity management. In addition, comprehensive and specific risk of bias assessment will be conducted for different study design. However, this review has potential limitations. The language of studies will be restricted to English and Chinese, meaning that evidence published in any other language will be missed.

## Author contributions

All authors read and approved the final manuscript.

**Conceptualization:** Wuyu Li, Hanghang Liu, Jie Wu.

**Data curation:** Yipeng Xu, Hanghang Liu.

**Investigation:** Maogui Yu.

**Methodology:** Yipeng Xu.

**Project administration:** Rui Luo, Jie Wu.

**Resources:** Zimeng Li.

**Software:** Zhengqi Pan, Hanghang Liu.

**Supervision:** Zimeng Li.

**Validation:** Shijie Huang.

**Visualization:** Shijie Huang, Wuyu Li, Hanghang Liu.

**Writing – original draft:** Shijie Huang.

**Writing – review & editing:** Shijie Huang.

## References

[1] Hope-Simpson RE. The nature of herpes zoster: a long-term study and a new hypothesis. Proc R Soc Med. 1965;58:9–20. 14267505 10.1177/003591576505800106 PMC1898279

[2] Black S. Herpes zoster vaccine and the medicare population. Clin Infect Dis. 2017;64:794–5. 28362956 10.1093/cid/ciw858

[3] Katz JE Cooper EM Walther RR. Acute pain in herpes zoster and its impact on health-related quality of life. Clin Infect Dis. 2004;39:342–48. 15307000 10.1086/421942

[4] Thompson RR Kong CL Porco TC. Herpes zoster and postherpetic neuralgia: changing incidence rates from 1994 to 2018 in the United States. Clin Infect Dis. 2021;73:e3210–7. 32829399 10.1093/cid/ciaa1185 PMC8563174

[5] Schmader KE Dworkin RH. The epidemiology and natural history of herpes zoster and postherpetic neuralgia. 2017:25–44.

[6] Weinke T Glogger A Bertrand I. The societal impact of herpes zoster and postherpetic neuralgia on patients, life partners, and children of patients in Germany. Sci World J. 2014;2014:749698. 10.1155/2014/749698 PMC4274846 25548792

[7] Robert H. Dworkin Robert W Johnson Judith Breuer. Recommendations for the management of herpes zoster. Clin Infect Dis. 2007;44(Suppl 1):1–26. 10.1086/510206 17143845

[8] Chen N Li Q Yang J. Antiviral treatment for preventing postherpetic neuralgia. Cochrane Database Syst Rev. 2014:CD006866. 24500927 10.1002/14651858.CD006866.pub3 PMC10583132

[9] Aydin T Balaban O Ahiskalioglu A. Ultrasound-guided erector spinae plane block for the management of herpes zoster pain: observational study. Cureus. 2019;11:e5891. 31772861 10.7759/cureus.5891 PMC6837261

[10] Haythornthwaite JA Clark MR Pappagallo M. Pain coping strategies play a role in the persistence of pain in post-herpetic neuralgia. Pain. 2003;106:453–60. 14659529 10.1016/j.pain.2003.09.009

[11] Ducic I Felder JM. Peripheral nerve surgery for the treatment of postherpetic neuralgia. Ann Plast Surg. 2013;71:384–85. 23528637 10.1097/SAP.0b013e318273f650

[12] Zhou Q Wei S Zhu H. Acupuncture and moxibustion combined with cupping for the treatment of post-herpetic neuralgia: a meta-analysis. Medicine (Baltim). 2021;100:e26785. 10.1097/MD.0000000000026785 PMC8341313 34397828

[13] Lee MS Ernst E. Acupuncture for pain: an overview of Cochrane reviews. Chin J Integr Med. 2011;17:187–9. 21359919 10.1007/s11655-011-0665-7

[14] Jiaxuan W Weixuan Z Jingchun Z. Systematic review and sequential analysis on treatment of herpes zoster pain mainly by fire needle therapy. Acupuncture Res. 2019;44:677–85. 10.13702/j.1000-0607.190004 31532139

[15] Wang Y Li W Peng W. Acupuncture for postherpetic neuralgia: systematic review and meta-analysis. Medicine (Baltim). 2018;97:e11986. 10.1097/MD.0000000000011986 PMC6113033 30142834

[16] Page MJ McKenzie JE Bossuyt PM. The PRISMA 2020 statement: an updated guideline for reporting systematic reviews. BMJ. 2021;372:n71. 33782057 10.1136/bmj.n71 PMC8005924

[17] Johnson RW. The future of predictors, prevention, and therapy in postherpetic neuralgia. Neurology. 1995;45:70–2. 10.1212/wnl.45.12_suppl_8.s70 8545029

[18] Gopalakrishna G Mustafa RA Davenport C. Applying grading of recommendations assessment, development and evaluation (GRADE) to diagnostic tests was challenging but doable. J Clin Epidemiol. 2014;67:760–8. 24725643 10.1016/j.jclinepi.2014.01.006

[19] Schmader KE. Epidemiology and impact on quality of life of postherpetic neuralgia and painful diabetic neuropathy. Clin J Pain. 2002;18:350–4. 12441828 10.1097/00002508-200211000-00002

[20] Decroix J Partsch H Gonzalez R. Factors influencing pain outcome in herpes zoster: an observational study with valaciclovir. J Eur Acad Dermatol Venereol. 2000;14:23–33. 10877249 10.1046/j.1468-3083.2000.00020.x

[21] Li W Peng W Zhou J. Acupuncture for postherpetic neuralgia: a systematic review protocol. BMJ Open. 2014;4:e005725. 10.1136/bmjopen-2014-005725 PMC4244400 25392023

